# The Therapeutic Effect of STAT3 Signaling-Suppressed MSC on Pain and Articular Cartilage Damage in a Rat Model of Monosodium Iodoacetate-Induced Osteoarthritis

**DOI:** 10.3389/fimmu.2018.02881

**Published:** 2018-12-11

**Authors:** Seon-yeong Lee, Seung Hoon Lee, Hyun Sik Na, Ji Ye Kwon, Goo-Young Kim, KyungAh Jung, Keun-Hyung Cho, Seon Ae Kim, Eun Jeong Go, Min-Jung Park, Jin-Ah Baek, Si Young Choi, JooYeon Jhun, Sung-Hwan Park, Seok Jung Kim, Mi-La Cho

**Affiliations:** ^1^The Rheumatism Research Center, Catholic Research Institute of Medical Science, College of Medicine, The Catholic University of Korea, Seoul, South Korea; ^2^Impact Biotech, Seoul, South Korea; ^3^Department of Orthopedic Surgery, Uijeongbu St. Mary's Hospital, College of Medicine, The Catholic University of Korea, Seoul, South Korea; ^4^Division of Rheumatology, Department of Internal Medicine, Seoul St. Mary's Hospital, College of Medicine, The Catholic University of Korea, Seoul, South Korea; ^5^Department of Biomedicine and Health Sciences, College of Medicine, The Catholic University of Korea, Seoul, South Korea; ^6^Department of Medical Life Science, College of Medicine, The Catholic University of Korea, Seoul, South Korea

**Keywords:** osteoarthritis, inflammation, mesenchymal stem cells, stat3, TRPV1

## Abstract

Osteoarthritis (OA) is a degenerative disease that induces pain, cartilage deformation, and joint inflammation. Mesenchymal stem cells (MSCs) are potential therapeutic agents for treatment of OA. However, MSC therapy can cause excessive inflammation. Signal transducer and activator of transcription 3 (STAT3) modulates secretion of many proinflammatory cytokines. Experimental OA was induced by intra-articular (IA) injection of monosodium iodoacetate (MIA) to the right knee of rats. MSCs from OA patients (OA-MSCs) were treated with STA21, a small molecule that blocks STAT3 signaling, by IA or intravenous (IV) injection after MIA injection. Pain severity was quantified by assessment of secondary tactile allodynia using the von Frey assessment test. Cartilage degradation was measured by microcomputed tomography image analysis, histological analysis, and the Mankin score. Protein and gene expression was evaluated by enzyme-linked immunosorbent assay, immunohistochemistry, and real-time polymerase chain reaction. MSCs increased production of proinflammatory cytokines under inflammatory conditions. STA21 significantly decreased expression of these proinflammatory molecules via inhibition of STAT3 activity but increased gene expression of molecules related to migration potential and immunomodulation in OA-MSCs. STAT3-inhibited OA-MSCs administrated by IV or IA injection decreased pain severity and cartilage damage in rats with MIA-induced OA rats by decreasing proinflammatory cytokines in the joints. Combined IA and IV-injected STAT3-inhibited OA-MSCs had an additive effect of pain relief in MIA-induced OA rats. STAT3 inhibition may optimize the therapeutic activities of MSCs for treating OA by attenuating pain and progression of MIA by inhibiting inflammation and cartilage damage.

## Introduction

Osteoarthritis (OA) is a common degenerative joint disease that is associated with pain and devastation of articular cartilage. Severe pain is the clinical hallmark of OA, which can impair quality of life. OA is also a highly prevalent cause of disability (1). OA is associated with several risk factors, including age and joint trauma, and is considered to be an inflammatory disease characterized by joint inflammation (2). The levels of several proinflammatory cytokines, such as interleukin 6 (IL-6) and tumor necrosis factor α (TNF-α) in plasma, cartilage, or synovial fluid are higher in OA patients than in healthy controls (3–5).

Mesenchymal stem cells (MSCs) are well-documented immune modulators that can limit the inflammatory response. Given these characteristics, several studies have suggested that MSCs have potential as therapeutic factors for regenerative cell therapy for OA (6). MSCs can differentiate into cartilage and exhibit immunosuppressive activity, and may provide a new approach for treating OA (7). However, MSC therapy has many problems because increasing evidence suggests that MSCs fail to exhibit immunoregulatory activity and may upregulate inflammation under certain conditions (8).

MSCs can be differentiated into several cells with a proinflammatory phenotype (9). MSC treatment fails to decrease the immune response in the inflammatory state associated with inflammatory arthritis (7). A recent study reported that MSCs provided limited benefits for OA because of their immunomodulatory functions (10). Therefore, optimization of MSC immunomodulatory function may be necessary before clinical applications in the treatment of OA.

Signal transducer and activator of transcription 3 (STAT3) is a DNA-binding molecule that regulates the levels of many cytokines. Activation of STAT3 leads to increased proinflammatory cytokine production and immune responses (11, 12). Recent reports have shown that inhibition of STAT3 improves experimental autoimmune arthritis by limiting the immune–inflammatory response (13–15). STAT3 activation has also been shown to be significantly higher in chondrocytes from OA patients than in those from normal controls (16). Taken together, these reports suggest that STAT3 inhibition may have therapeutic potential for treatment of inflammatory diseases.

We hypothesized that inhibition of STAT3 signaling (iSTAT3) in MSCs derived from adipose tissue of OA patients (OA-MSCs) would augment the immunomodulatory functions of OA-MSCs. To optimize the therapeutic potential of MSCs in the treatment of OA, we treated OA-MSCs with the STAT3 inhibitor STA21 (hereafter, these STA21-treated OA-MSCs are referred to as iSTAT3-OA-MSCs). The anti-inflammatory activity and differentiation potential of iSTAT3-OA-MSCs was evaluated *in vitro*. The *in vivo* therapeutic potential of iSTAT3-OA-MSCs was investigated in the monosodium iodoacetate (MIA)-induced rat model of OA.

## Materials and Methods

### Animals

Six-week old male Wistar rats (Central Lab. Animal Inc., Seoul, Korea) weighing ~190 g at the start of the experiment were purchased from Central Lab Animal Inc. (Seoul, South Korea). The animals were housed 3 per cage in a room with controlled temperature conditions (21–22°C) and lighting (12-h light/dark cycle) with access to sterile food and water. The animal used three per each group and the each repeated six independent experiment for *in vivo* efficacy. All experimental procedures were examined and approved by the Animal Research Ethics Committee of The Catholic University of Korea (2016-0060-04) and conformed to the National Institutes of Health guidelines.

### Study Population

The samples were collected from 19 patients with OA. OA patient were recruited from the Orthopedic Surgery, Uijeongbu St. Mary's Hospital, Seoul, Korea (IRB NO. UC14CNSI0150). All the cased fulfilled the 1986 American College of Rheumatology (ACR) criteria for the classification and reporting of osteoarthritis of the knee. All procedures performed in this study were in accordance with the ethical standards of our Institute and with the 1964 Helsinki declaration.

### Isolation and Culture of OA-MSCs

Adipose tissues were collected from OA patients and were digested with a sterile scissors for 2 min. The tissues were digested with type I collagenase (Cat. LS004197; Worthington Biochemical Products, Lakewood, NJ, USA) at 37°C in a water bath for 40 min and then centrifuged at 1,500 rpm for 5 min. The pellet was resuspended in Dulbecco's phosphate-buffered saline (PBS) and filtered through a 40-μM strainer. Cells were resuspended and cultured with Dulbecco's modified Eagle's medium containing 10% fetal bovine serum (Pan-Biotech, Aidenbach, Germany). The cells were cultured for 4 days until 90% confluent (passage 0) and were then expanded for 2–3 passages and used for experiments. iSTAT3 OA-MSCs were prepared by treatment of OA-MSCs with 10 μM STA21 (Santa Cruz, Texas, USA) for 72 h.

### Induction of OA and Treatment of iSTAT3-OA-MSCs

Animals were randomly assigned to treatment groups before the study began. After anesthetization with isoflurane, rats were injected with 3 mg of MIA (Sigma-Aldrich, St. Louis, MO, USA) in a 50-μl volume using a 26.5-G needle inserted through the patellar ligament into the intra-articular (IA) space of the right knee. Control rats were injected with an equivalent volume of PBS. Each rat was injected twice with 3 × 10^5^ OA-MSCs and iSTAT3 OA-MSCs via IA, IV, IA, and IV administration at day 1 and 5 after MIA injection.

### Assessment of Pain Behavior

MIA-treated rats were randomized to experimental groups. Nociception was tested using a dynamic plantar esthesiometer (Ugo Basile, Gemonio, Italy), which is essentially an automated version of the von Frey hair assessment procedure used to assess mechanical sensitivity. Rats were placed on a metal mesh surface in an acrylic chamber in a temperature-controlled room (21–22°C) and rested for 10 min before testing. The touch stimulator unit was positioned under the animal. An adjustable angled mirror was used to place the stimulating microfilament (0.5-mm diameter) below the plantar surface of the hind paw. When the instrument was activated, a fine plastic monofilament advanced at a constant speed and touched the paw in the proximal metatarsal region. The filament exerted a gradually increasing force on the plantar surface, which started below the threshold of detection and increased until the stimulus became painful, as indicated by removal of the paw. The force required to elicit a paw-withdrawal reflex was recorded automatically and measured in g. A maximum force of 50 g and a ramp speed of 25 s were used for all esthesiometry tests. Pain behavioral tests of secondary tactile allodynia were conducted immediately before injection of OA-MSCs or iSTAT3 OA-MSCs.

### Real-Time Polymerase Chain Reaction

The relative expression of specific messenger RNA (mRNA) was quantified by real-time polymerase chain reaction (PCR) using SYBR Green I (Roche Diagnostics, Mannheim, Germany). The following sense and antisense primers were used: for IL-6, 5′-TGA GGA GAC TTG CCT GGT GAA-3′, reverse 5′-CAG CTC TGG CTT GTT CCT CAC-3′; for IL-8, 5′-GAG AGT GAT TGA GAG TGG ACC AC-3′, reverse 5′-CAC AAC CCT CTG CAC CCA GTT T-3′; for IL-10, 5′-CCA AGC CTT GTC TGA GAT GA-3′, reverse 5′-TGA GGG TCT TCA GGT TCT CC; for IL-1β, 5′-GGA CAA GCT GAG GAA GAT GC-3′, reverse 5′-TCG TTA TCC CAT GTG TCG AA-3′; for VEGF, 5′-CCA TGA ACT TTC TGC TGT CTT-3′, reverse 5′-ATC GCA TCA GGG GCA CAC AG-3′; for transforming growth factor-β (TGF-β), 5′-TGC GGC AGC TGT ACA TTG A-3′, reverse 5′-TGG TTG TAC AGG GCC AGG A-3′; for C-C chemokine receptor type 2 (CCR2), 5′-CTA CCT TCC AGT TCC TCA TTT T-3′, reverse 5′-ACA TTT ACA AGT TGC AGT TTT CAG C-3′; for CCR3, 5′-TTT GTC ATC ATG GCG GTG TTT TTC-3′, reverse 5′-GGT TCA TGC AGC AGT GGG AGT AG-3′; for CCR9, 5′-TAT ACA GCC AAA TCA AGG AAT C-3′, reverse 5′-CAT GAC CAC GAA GGG AAG GAA G-3′ and for β-actin 5′- GGA CTT CGA GCA AGA GAT GG-3′, reverse 5′- TGT GTT GGG GTA CAG GTC TTT G-3′.

### Western Blot Analysis

Proteins were separated by sodium dodecyl sulfate-polyacrylamide gel electrophoresis and transferred onto nitrocellulose membranes (Amersham Pharmacia Biotech, Piscataway, NJ, USA). Western blotting was performed using a SNAP i.d. Protein Detection System (Millipore, Billerica, MA, USA). The hybridized bands were detected using an enhanced chemiluminescence detection kit (Thermo Fisher Scientific, Waltham, MA, USA). The antibodies were as follows: phospho-STAT3 705, phospho-STAT3 727, Total STAT3 (Cell Signaling Technologies, Danvers, MA, USA), β-actin, and goat horseradish peroxidase-conjugated anti-rabbit IgG.

### Enzyme-Linked Immunosorbent Assay

The concentrations of IL-6, IL-8, IL-1β, and vascular endothelial growth factor (VEGF) were measured in culture supernatants of OA-MSCs using a sandwich enzyme-linked immunosorbent assay (ELISA) kit (DuoSet; R&D Systems, Lille, France). For measurement of IL-6, IL-8, IL-1β, and VEGF, each specific Abs were coated to a 96-well plate and incubated overnight at 4°C, and this was followed by a blocking step. The culture supernatants of OA-MSCs and standard of each cytokine were added to plate and incubated in room temperature for 2 h. Biotinylated polyclonal cytokine specific Abs was added and the reaction was allowed to proceed. The plate was washed, and reaction of ExtrAvidin-alkaline phosphatase (Sigma Aldrich, Louis, USA). The absorbance values were determined with ELISA microplate reader operating at 405 nm.

### Flow Cytometry Analysis

Monoclonal antibodies (mAbs) conjugated to fluorescent dyes targeting human CD13, CD90, CD105, CD29, CD44, CD11b, CD19, CD31, CD34, and human leukocyte antigen—antigen D-related (HLA-DR) (BD Biosciences, Schwechat, Austria) were used to characterize OA-MSCs. The cell surface was stained using different combinations of the following mAbs (all from Pharmingen, San Diego, CA, USA, unless indicated): CD13–phycoerythrin (PE) (L138, immunoglobulin 1 [IgG1], κ); CD90–PE (5E10, IgG1, κ); CD11b–PE (ICRF44, IgG_1_, κ); CD19–PE (HIB19, IgG1, κ); CD34–PE (8G12, IgG1, κ); CD34–fluorescein isothiocyanate (FITC) (MMA, IgM); CD105–allophycocyanin (APC) (RPA-T4, IgG1; BioLegend, San Diego, CA, USA); CD29–PE (RPA-T8, IgG1, κ); CD44–FITC (HI100, IgG2b, κ); CD45–APC (M-A251, IgG1, κ); and HLA-DR–PE (G46-6, IgG2a, κ). The data were analyzed using FlowJo software (Tree Star, Ashland, OR, USA).

### Evaluation of MSC Differentiation

OA-MSCs were plated at 2 × 10^4^ cells/well in a 6-well plate and cultured in basal complete culture medium until the cells reached 90–100% confluence and then incubated in osteogenic and adipogenic medium (A1007201, 1007001; Gibco, Fisher Scientific, UK). The medium was replaced every 2–3 days. On day 14, lipid droplet and calcium nodule formation were evaluated using Oil red O staining and Alizarin red/alkaline phosphatase (ALP) staining, respectively. Chondrogenic differentiation was assessed by the pellet culture method as modified recently. A total of 2.5 × 10^5^ cells were centrifuged at 450 g for 10 min to form a micromass in a 15-ml conical polypropylene tube and cultured in chondrogenic medium (cat. no. A1007101; Gibco) at 37°C with 5% CO_2_. On day 21, the pellet was fixed with 10% formalin and then embedded in paraffin. Safranin O, Alcian blue (pH 2.5), and type II collagen staining were used to examine glycosaminoglycan deposition and type II collagen.

### Histopathology Analysis

Rat joint tissues were obtained 4 weeks after MIA injection. Joint tissues were sectioned at 5-μm thickness, dewaxed using xylene, dehydrated through an alcohol gradient and stained with hematoxylin and eosin (H&E) and Safranin O. Each knee joint tissue for grading assessment is limited to articular surface and femoral chondyle. Articular cartilage OA histopathology of Rat was assessed using the OARSI and Mankin scoring methods (17, 18). The all confocal and IHC images were obtained each mouse, and showing representative images. The percentage of positive cells showing in each image was measured.

### Immunohistochemistry

Paraffin samples were incubated at 4°C with the first primary mAb. The mAbs used were TNF-α, IL-1β, IL-6, hypoxia-inducible factor 2α (HIF-2α) (all from abcam, MA, USA), and transient receptor potential cation channel subfamily V member (TRPV)-1 (R&D Systems). The samples were incubated with the appropriate biotinylated secondary antibody and then incubated with streptavidin–peroxidase complex for 1 h. The product was developed using 3,3′-diaminobenzidine chromogen (Dako, Carpinteria, CA, USA). Positive cells were counted, and the results are expressed as mean ± standard deviation (SD).

### *In vivo* Microcomputed Tomography Imaging and Analysis

Microcomputed tomography (micro-CT) imaging and analysis were performed using a benchtop cone-beam type *in vivo* animal scanner (SkyScan1172 micro-CT; Bruker Micro CT, Brussels, Belgium). Briefly, 2 weeks after the injection, rats were sacrificed with CO_2_ euthanasia. The hind limbs were dissected and immediately fixed in 10% neutral buffered formalin. Samples were imaged with settings of 70 kVp and 141 μA, and with an aluminum 0.5-mm thick filter. The pixel size was 8.0 μm and the rotation step was 0.4°. The cross-sectional images were reconstructed using a filtered back-projection algorithm (NRecon software; Bruker Micro CT). For each scan, a stack of 286 cross-sections was reconstructed at 2,000 × 1,335 pixels. Bone mineral density was calculated at the lateral femoral condyle area of each sample. A semiquantitative method using the degree of osteophytes and cartilage surface was introduced to grade the degree of OA changes.

### Statistical Analysis

Statistical analysis performed using GraphPad Prism software (version 5.01, GraphPad Software, San Diego, CA, USA). Comparisons of numerical data among the three or four groups were performed with 2-say ANOVA and non-parametric Mann–Whitney *U*-test and difference in the mean value of various groups using ANOVA with *post hoc* test. The *p* values < 0.05 were considered to be significant. Data are presented as means ± standard deviation.

## Results

### STAT3 Inhibition Induced Anti-inflammatory Activity and Migration Potential in OA-MSCs

We isolated MSCs from adipose tissues of OA patients (OA-MSCs) and confirmed that the cells showed positive and negative markers of MSCs (Supplementary Figure 1). We also check their ability to differentiate into multiple lineages, including adipocytes, osteocytes, and chondrocytes (Supplementary Figure 2). Next, we treated OA-MSCs with various inflammatory mediators and used ELISA to measure the concentrations of proinflammatory cytokines. Significantly higher concentrations of proinflammatory cytokines (IL-6, IL-8, IL-1β, and VEGF) were measured in the culture supernatant from OA-MSCs treated with inflammatory mediators than in that from unstimulated OA-MSCs (Supplementary Figure 3). We also measured that how long injected OA-MSCs can survive in rat. The cells were detected in rat until 12 days after injection (Supplementary Figure 4).

**Figure 1:**
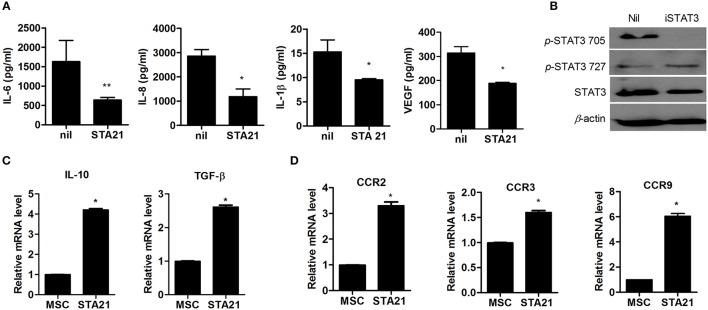
Effects of STAT3 inhibition on anti-inflammatory activity and migration potential in MSCs from osteoarthritis (OA) patients. **(A)** OA-MSCs were incubated with STA21 for 3 days and, ELISA was performed to measure IL-6, IL-8, IL-1β, and VEGF concentrations. **(B)** OA-MSCs were incubated with STA21 for 1 h. The expression of phospho (p)-STAT3 705, 727, and total STAT3 protein was analyzed by Western blotting. **(C)** mRNA levels for anti-inflammatory cytokines IL-10 and TGF-β were measured by real time-PCR **(D)** Expression of chemokine receptors in OA-MSCs incubated with STA21 was measured by real time-PCR. OA-MSC were isolated of fat tissues obtain from each 3 person with OA. Data represent the mean ± SD of 3 independent experiments (^*^P < 0.05; ^**^p < 0.01).

To examine whether inhibition of STAT3 signaling can regulate the expression of proinflammatory cytokines in OA-MSCs, OA-MSCs were treated with the STAT3-selective inhibitor STA21, producing iSTAT3 OA-MSCs. The concentrations of proinflammatory cytokines was decreased significantly in iSTAT3 OA-MSCs (Figure 1A). The concentration of phospho-STAT3 705, but not phospho-STAT3 727, was also reduced in iSTAT3-OA-MSCs, (Figure 1B).

We performed real-time PCR to assess anti-inflammatory function and migration potential in iSTAT3 OA-MSCs. The transcript levels of anti-inflammatory mediators and chemokine receptors were increased significantly by STA21 (Figures 1C,D). These data suggest that MSCs under the inflammatory milieu in the OA joint may increase the expression of inflammatory mediators, which may relate to the lack of therapeutic activity *in vivo*. Therefore, iSTAT3 OA-MSCs may increase the anti-inflammatory response and therapeutic potential for treating OA.

### IA or IV Administration of STAT3-Inhibited OA MSCs Reduced the Severity of OA in MIA-Induced OA Rats

**Figure 2:**
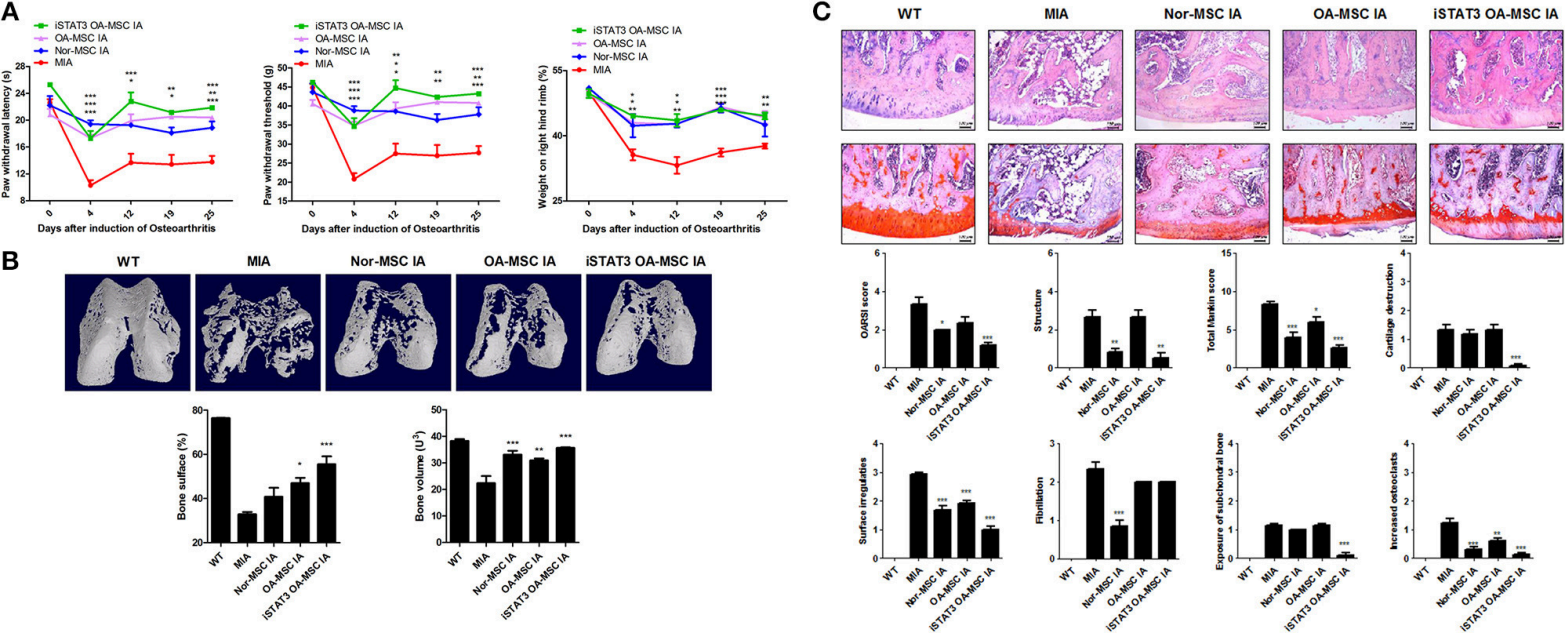
Reduced severity by IA administration of iSTAT3 OA MSCs in MIA-induced OA rats. **(A)** OA was induced in Wistar rats by IA injection of MIA. OA Rats were injected with IA of normal (Nor-) MSCs (6 × 10^5^), OA-MSCs (6 × 10^5^), or iSTAT3 OA-MSCs (6 × 10^5^). Nor-MSC were isolated of fat tissues obtain from each 2 normal person, and OA-MSC were from each 4 person with OA. Pain behavior was assessed as mechanical hyperalgesia measured with a dynamic plantar esthesiometer and incapacitance meter, and was quantified as PWL and PWT (*n* = 3 per group). **(B)** Representative micro-CT images of the femoral condyles at 4 weeks in all groups. Object volume (Obj.V) and bone surface (%) were measured in micro-CT images from femurs. (^*^P < 0.05, ^**^P < 0.01, ^***^P < 0.001). **(C)** Histochemical analysis of Femoral condyle and severity scores after IA administration of iSTAT3-OA MSCs to MIA-induced OA rats. Knee joint tissue samples were acquired from all OA groups at 4 weeks and stained with H&E and Safranin O to evaluate the severity of inflammation and cartilage damage (^*^P < 0.05, ^**^P < 0.01, ^***^P < 0.001).

**Figure 3:**
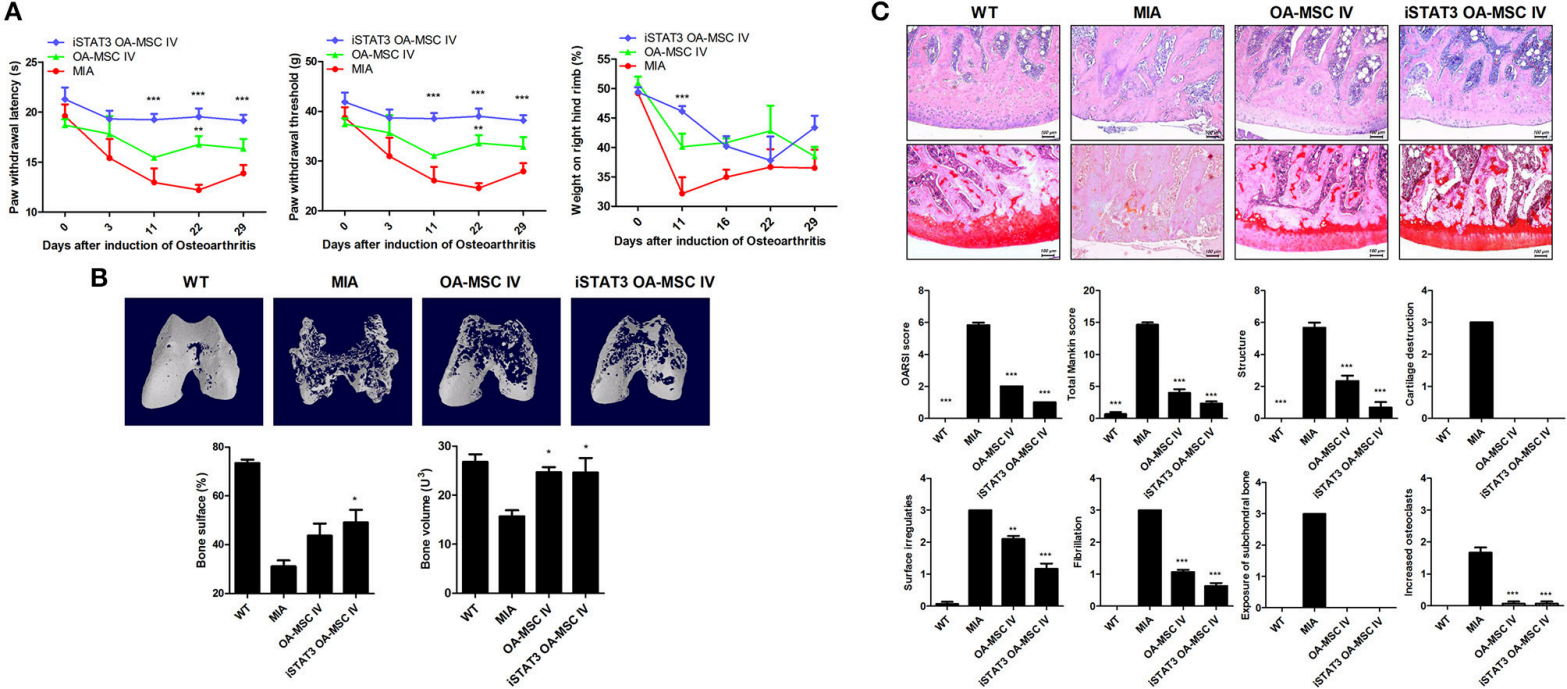
Reduced severity by IV administration of iSTAT3 OA-MSCs in MIA-induced OA rats. **(A)** OA was induced in Wistar rats by IA injection of MIA. OA rats were injected IV with OA-MSCs (6 × 10^5^) or iSTAT3 OA-MSC (6 × 10^5^). OA-MSC were isolated of fat tissues obtain from each 3 person with OA. Pain behavior was assessed as mechanical hyperalgesia measured with a dynamic plantar esthesiometer and incapacitance meter, and was quantified as PWL and PWT (*n* = 3 per group). **(B)** Representative micro-CT images of the femoral condyles at 4 weeks in all groups. Object volume (Obj.V) and bone surface (%) measured in micro-CT images from femurs. **(C)** Histochemical analysis of femoral condyle and severity scores after IV administration of iSTAT3-OA MSCs in MIA-induced OA rats. Knee joint tissue samples were acquired at 4 weeks from wild-type (WT) rats, rats with MIA-induced OA, rats that received IV OA-MSCs, and rats that received IV iSTAT3 OA-MSCs, and were stained with H&E and Safranin O to evaluate the severity of inflammation and cartilage damage (^*^P < 0.05; ^**^P < 0.01; ^***^P < 0.001).

Secondary tactile allodynia was measured in MIA-induced OA rats to assess the therapeutic activity of iSTAT3 OA-MSCs. IA or IV treatment of iSTAT3 OA-MSCs promoted paw-withdrawal latency (PWL) and paw-withdrawal threshold (PWT) in MIA-induced OA rats compared with normal MSCs or OA-MSCs. Weight bearing was also increased by IA or IV injection of iSTAT3 OA-MSCs in OA rats (Figures 2A, 3A).

We performed micro-CT to analyze joints from MIA-induced OA rats to determine whether iSTAT3 OA-MSCs exert protective activity in the femur. IA or IV administration of iSTAT3-OA MSCs seemed to have protected the femurs in MIA-induced OA rats (Figures 2B, 3B). The object volume (Obj.V) and total surface (%) of the cartilage region of the femur were greater in rats that received IA or IV administration of iSTAT3 OA-MSCs compared with those that received normal MSCs or OA-MSCs (Figures 2B, 3B). IA or IV administration of iSTAT3 OA-MSCs also attenuated cartilage damage and proteoglycan content compared with those of rats that received normal MSCs or OA-MSCs (Figures 2C, 3C). The histological scores were lower in rats that received iSTAT3 OA-MSCs than in those that received normal MSCs or OA-MSCs. These results suggest that, in the local inflammatory milieu, MSCs may exert weak therapeutic activity that attenuates OA progression, whereas STAT3 inhibition of OA MSCs may optimize their therapeutic activity.

### IA and IV Administration of STAT3-Inhibited OA-MSCs Had an Additive Effect of Pain Relief in MIA-Induced OA Rats

**Figure 4:**
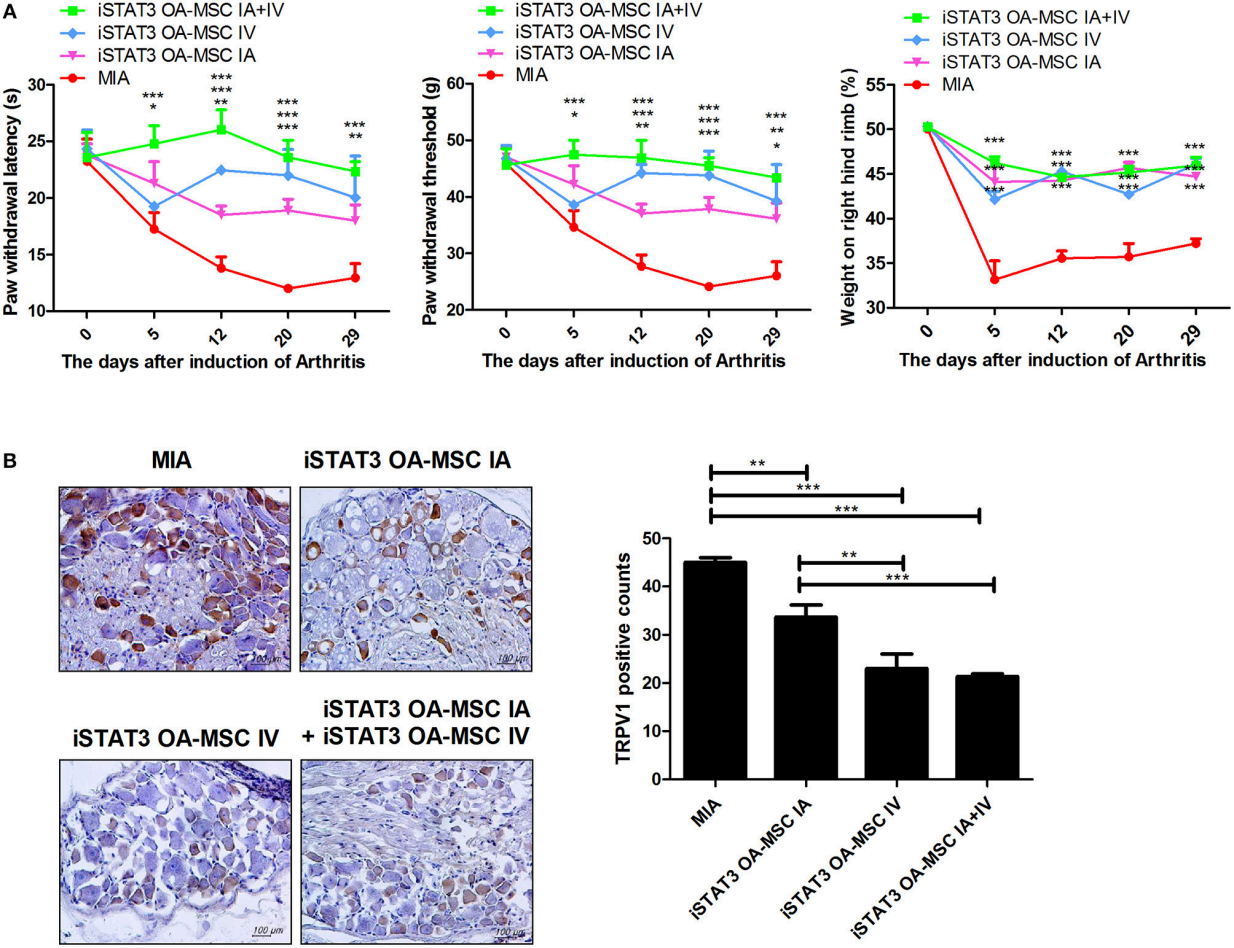
Additive effects of pain relief by IA and IV co-administration of iSTAT3 OA-MSCs in MIA-induced OA rats. **(A)** OA was induced in Wistar rats by IA injection of MIA. OA rats were injected with IA and IV iSTAT3 OA-MSCs (each 3 × 10^5^, final 6 × 10^5^ per rat). OA-MSC were isolated of fat tissues obtain from each four person with OA. Pain behavior was assessed as mechanical hyperalgesia measured with a dynamic plantar esthesiometer and incapacitance meter, and was quantified as PWL and PWT (*n* = 3 per group). **(B)** Immunohistochemical staining was used to identify the expression of TRPV1 in the dorsal root ganglion of OA rats. ^*^P < 0.05, ^**^P < 0.01, ^***^P < 0.001.

We next investigated whether IA and IV co-administration of iSTAT3 OA-MSCs would have a synergetic effect in MIA-induced OA rats. Combined IA and IV administration of iSTAT3 OA-MSCs improved nociceptive pain severity, as shown by the higher PWL and PWT, compared with IA or IV administration of iSTAT3 OA-MSCs alone (Figure 4A). The expression of TRPV1, which is related to pain, was reduced by IA and IV co-administration of iSTAT3-OA MSCs (Figure 4B). Although the joint-protective effect was lower for IV administration than for IA administration (Figures 2, 3), the expression levels of TRPV1 were lower after IV than after IA administration (Figure 4B). These findings suggest that the pain-relieving effect was more closely related to IV administration of iSTAT3-OA MSCs than to IA administration of iSTAT3-OA MSCs.

### IA and IV Administration of STAT3-Inhibited OA-MSCs Had a Additive Effect on Joint Protection in MIA-Induced OA Rats

**Figure 5:**
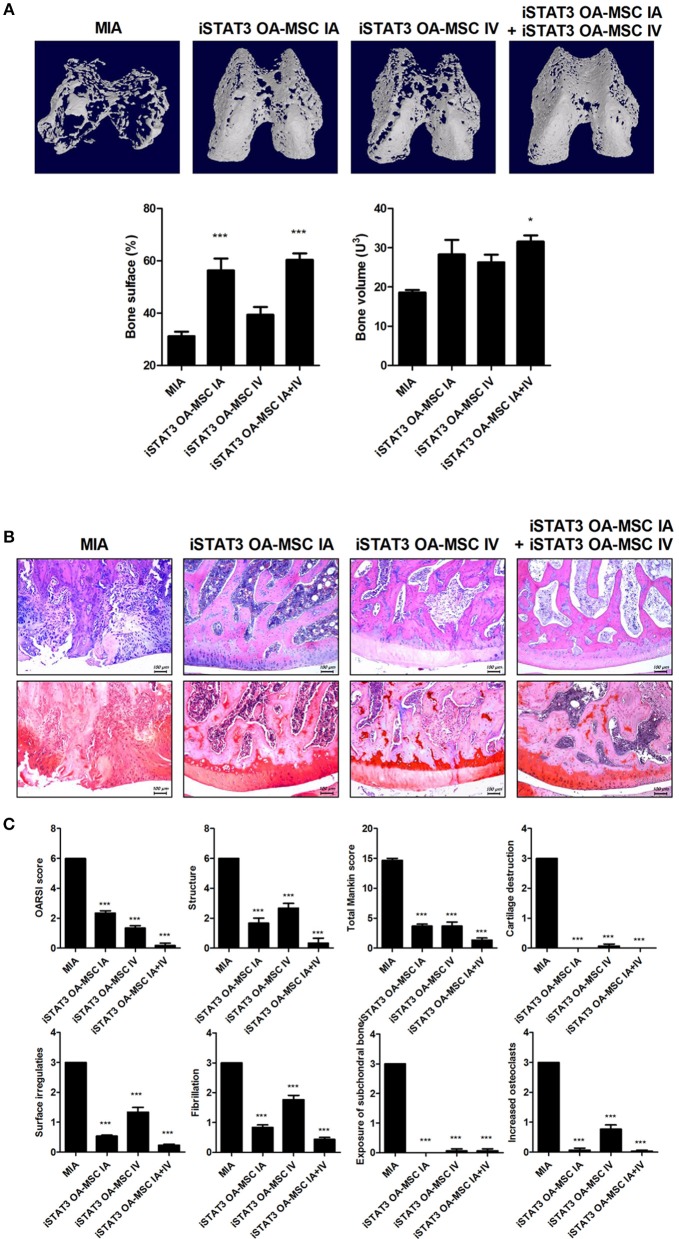
Additive effect of IA and IV co-administration of iSTAT3 OA-MSCs on joint protection in MIA-induced OA rats. **(A)** Representative micro-CT images of the femoral condyles at 4 weeks in all groups. Object volume (Obj.V) and bone surface (%) from micro-CT images of the femur (^*^P < 0.05, ^***^P < 0.001). **(B,C)** Histochemical analysis of femoral condyle **(B)** and severity scores **(C)** after IA and IV co-administration of iSTAT3-OA MSCs in MIA-induced OA rats. **(B)** OA was induced in Wistar rats by IA injection of MIA. OA Rats were injected multiple times with IA and IV co-administration of iSTAT3 OA-MSCs (3 × 10^5^). Knee joint tissue samples were acquired at 4 weeks from wild-type (WT) rats, rats with MIA-induced OA, rats that received IV OA-MSCs, and rats that received IV iSTAT3 OA-MSCs, and were stained with H&E and Safranin O to evaluate the severity of inflammation and cartilage damage (^***^P < 0.001).

**Figure 6:**
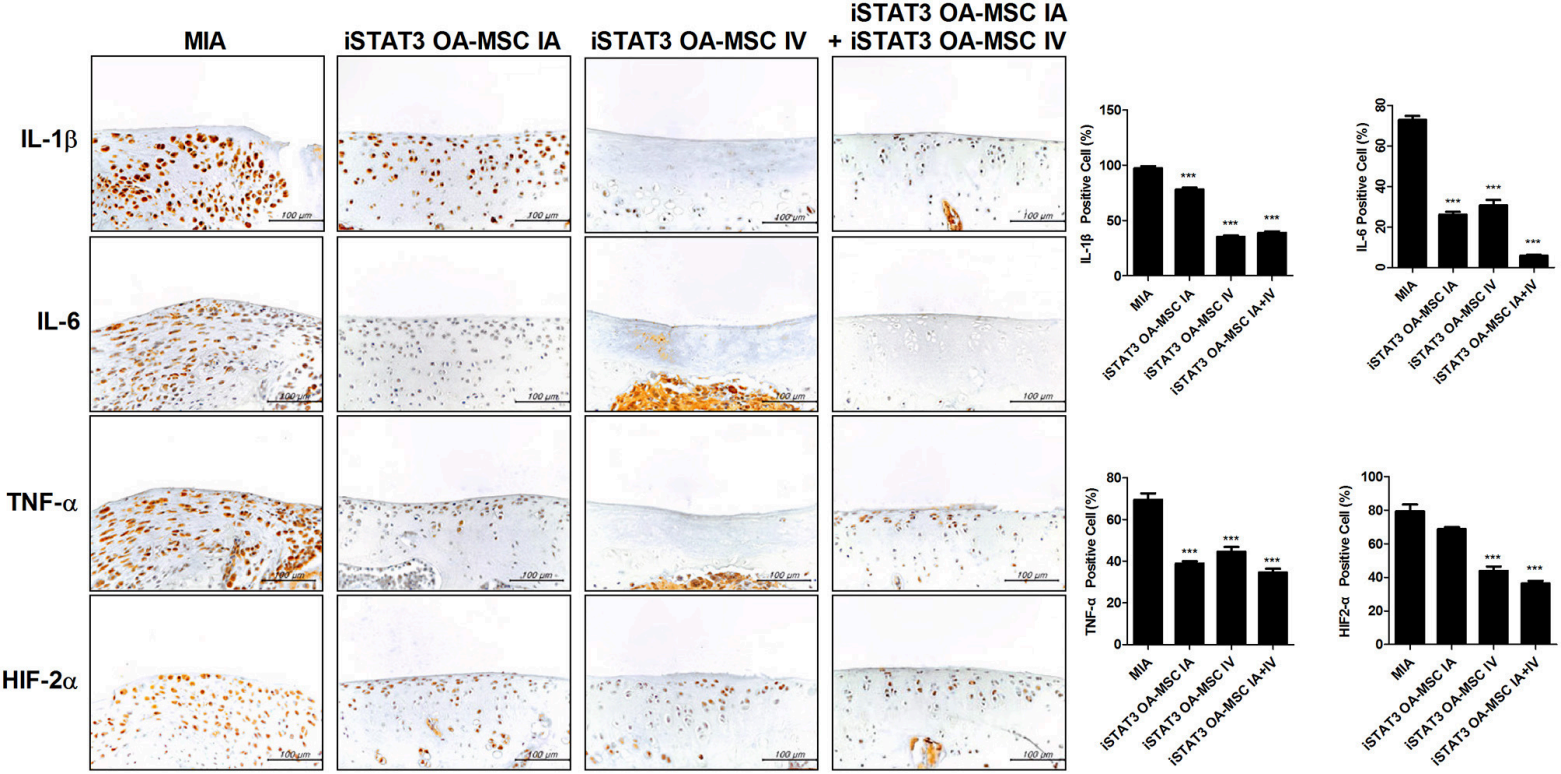
Expression of inflammatory mediators by administration of STAT3-inhibited OA-MSCs in articular cartilage of MIA-induced OA rats. Immunohistochemical staining of femoral condyle was performed to measure the expression of IL-1β, IL-6, TNF-α, and HIF-2α after multiple injections of IA and IV iSTAT3 OA-MSCs in knee joint tissues of MIA-induced OA rats. The percentage positive cells for each antibody are shown at the right. Data represent the mean ± SEM of 3 independent experiments (^***^P < 0.001).

Next, we assessed whether combined IA and IV injection of iSTAT3 OA-MSCs would have an additive effect on joints in MIA-induced OA rats. Co-administration of IA and IV iSTAT3 OA-MSCs had a protective effect by increasing the Obj.V and total surface % of the cartilage region of femurs in MIA-induced OA rats compared with IA or IV administration of iSTAT3 OA-MSCs alone (Figure 5A). Similarly, IA and IV co-administration of iSTAT3 OA-MSCs also resulted in less cartilage destruction and lower histological scores in MIA-induced OA rats than IA or IV administration of iSTAT3 OA-MSCs alone (Figures 5B,C). IA administration of iSTAT3 OA-MSCs induced greater protection against cartilage destruction and higher histological scores than IV administration (Figures 5A–C). In addition, the expression of inflammatory mediators was lowest in articular cartilage after co-administration of IA and IV iSTAT3 OA-MSCs in MIA-induced OA rats compared with the other groups (Figure 6). Taken together, these data suggest that IA and IV co-administration of iSTAT3 OA-MSCs may have an additive effect by slowing OA progression through the suppression of nociceptive pain severity, joint inflammation, and cartilage destruction.

## Discussion

Although MSCs have beneficial effects, including anti-inflammatory activity, in inflammatory disorders and several preclinical studies have raised concerns about their therapeutic applications (7, 19), MSC therapy for OA is limited by the possible proinflammatory phenotype (7, 9, 10). Therefore, blockade of inflammatory signaling in MSCs is needed before these cells can be used as cell therapy for OA. STAT3 is a prominent target for developing new treatments for inflammatory diseases (20, 21), but the biological activities associated with STAT3 inhibition in MSCs remain largely unknown. The main finding of the present study is that MSCs could be optimized by STAT3 inhibition in this rat model of experimental OA. We found that expression of proinflammatory cytokines decreased and that of anti-inflammatory mediators increased significantly in iSTAT3 OA-MSCs. We also observed that iSTAT3-OA MSCs reduced the severity of MIA-induced experimental OA in rats. Notably, IA and IV co-administration of iSTAT3-OA MSCs had an additive effect by attenuating pain severity and development of experimental OA compared with IA or IV administration alone. To our knowledge, this is the first study to suggest that STAT3 inhibition in OA-MSCs may optimize the immunotherapeutic activity of these cells. This may provide evidence for developing a new remedial approach for OA therapy.

OA has traditionally been recognized as a cartilage-degenerative and non-inflammatory arthritis. However, the difference between inflammatory and degenerative arthritis is becoming less apparent with the recognition of an excessive immune–inflammatory response within the OA joint and synovium (3–5). It has been suggested that proinflammatory cytokines accelerate pain and joint destruction and are involved in the pathogenesis of OA (22). Neutralizing proinflammatory cytokines can attenuate OA development by inhibiting of joint inflammation (23, 24). In the present study, administration of iSTAT3-OA MSCs was associated with downregulated expression of proinflammatory cytokines in the joints of MIA-induced rats. These results suggest that STAT3-inhibited OA-MSCs reduced inflammation in OA joints and may lead to the development of a novel cellular therapy for OA.

Because MSCs have immunosuppressive functions in inflammatory disorders and participate in immune–inflammatory processes, reduction in inflammation induced by MSCs may provide therapeutic activity. MSCs are considered to be a potential source of cells for OA treatment, but naïve MSCs fail to exert immunosuppressive and therapeutic activities *in vivo* (25). In a recent report, inflammatory signaling-suppressed MSCs decreased proinflammatory cytokine expression and had a therapeutic effect in experimental inflammatory arthritis (26). By contrast, anti-inflammatory mediators such as indoleamine 2,3-dioxygenase, IL-10, and TGF-β can exert immunosuppressive functions along with therapeutic activity in inflammatory diseases (27–29). Therefore, inhibition of MSC-induced inflammation may be crucial for the use of MSCs in OA therapy.

We observed that iSTAT3-OA MSCs downregulated inflammation but increased migration potential. Recently, it is reported that STAT3 inhibition can improve experimental OA development (30). IA and IV administration of iSTAT3-OA MSCs attenuated pain severity and experimental OA development compared with that induced by administration of normal and OA-MSCs. These findings suggest that STAT3-inhibited OA-MSCs may have a therapeutic function in the MIA-induced OA rat model via optimizing the immunosuppressive capacity. These results suggest that autologous MSCs from the inflammatory milieu of the OA joint may have clinical applications for the treatment of OA after restoration of their therapeutic activity by inhibition of STAT3 signaling.

OA development has been shown to correlate with pain severity. In a recent study, the radiographic grade of knee OA was significantly related to the level of knee pain (31). Pain caused by OA may involve both inflammatory and neuropathic pain originating in the synovial tissue. Upregulated expression of inflammatory mediators including TNF-α and IL-1β increases pain severity during the early stages of OA (32). Moreover, exacerbated fibrosis in the synovium and the upregulated production of neuromediators and chemokines both contribute to neuropathic pain during the late stages of OA (33–35). In this study, administration of STAT3-inhibited OA-MSCs reduced the expression of inflammatory mediators and chemokines in the cartilage region. Pain severity and TRPV1 production in the dorsal root ganglion were also decreased by injection of STAT3-inhibited OA MSCs in MIA-induced OA rats.

Interestingly, IV administration of iSTAT3-OA MSCs was more effective in reducing pain in MIA-induced OA rats. Previous studies have shown that IV administration of MSCs reduces the pathological severity and immune responses in several diseases, including rheumatoid arthritis (26), colitis (36), ischemia (37), and liver transplantation (38). These findings have led to speculation that pain relief by IV administration of iSTAT3-OA MSCs reflects systemic immunoregulatory effects on inflammation caused by OA. In addition, IA administration of iSTAT3 OA-MSCs induced greater protection against cartilage destruction and histological scores compared with IV administration, which suggests that direct IA administration increases the therapeutic concentration in the joint in MIA-induced OA rats. We also found that combined IV and IA administration of iSTAT3-OA MSCs significantly reduced the pathological severity compared with IV or IA administration alone in MIA-induced OA rats, even if total injected their MSCs number is identical. These results suggest that STAT3 inhibition in MSCs optimizes their immunoregulatory properties and that combined IV and IA administration of MSCs provides significantly better results in terms of reducing the severity of OA. These findings provide evidence for further studies of the potential use of MSCs in the treatment of OA.

The current study provides the first evidence that STAT3-suppression in MSCs may be optimized to improve OA. We believe that this may provide a strategy for optimizing the therapeutic potential of MSCs to control the inflammatory milieu and to limit OA progression. Downregulation of inflammatory responses in MSCs may be optimized to augment their clinical applications for OA treatment. Further refinement of the immunosuppressive activity of STAT3-suppressed MSCs identified in this study may lead to potential new clinical applications of MSCs for cell therapy for OA.

## Author Contributions

S-YL, SHL, HSN, and MLC designed the experiments, analyzed the data. S-YL, HSN, and SHL wrote the manuscript along with input from GYK, and MJP. SHL, and HSN performed all *in vitro* assays with help from JYK, JAB, and SYC. HSN, KHC, and JYK performed animal experiments. KAJ, EJG, and SAK conducted all immunohistochemistry experiments. SHP, SJK, and MLC discussed and developed the concept. All authors critically reviewed and approved the final form of the manuscript.

### Conflict of Interest Statement

The authors declare that the research was conducted in the absence of any commercial or financial relationships that could be construed as a potential conflict of interest.

## Supplementary Material

Supplementary Figure 1 Expression levels of MSC surface markers in OA-MSCs. OA-MSCs were isolated from adipose tissue from OA patients. Positive and negative surface markers on OA-MSCs were analyzed by flow cytometry. From left to right: upper row, hematopoietic cell markers CD11b, CD19, CD31, CD34, and HLA-DR; lower row, MSC surface markers CD90, CD13, CD44, CD29, and CD105. Red histograms indicated cells stained with the isotype control; blue histograms indicate cells isolated with specific antibodies.

Supplementary Figure 2 Multipotency of OA-MSCs and iSTAT3 OA-MSCs. MSCs can differentiate into a variety of connective tissues cells, including bone, cartilage, and adipose tissue. To observe this multipotent ability, OA-MSCs and iSTAT3 OA-MSCs were plated at 2 × 10^4^ cells/well in a 6-well plate and cultured in osteogenic adipogenic differentiation medium. On day 14, calcium nodule formation was evaluated using Alizarin red and ALP staining, and oil droplets were identified using Oil red O staining. Pellets were cultured to assess MSC chondrogenic differentiation. After 21 days of culture in chondrogenic differentiation medium, pellets were fixed in 10% neutral formalin and embedded in paraffin. The paraffin blocks were sliced at a thickness of 5 μm and stained with H&E, Alcian blue, type II collagen and Oil red O for immunohistochemical analysis.

Supplementary Figure 3 Inflammatory cytokine expression by OA-MSCs under inflammatory conditions. OA-MSCs were stimulated by proinflammatory cytokines IL-6, TNF-α, IL-1β, or lipopolysaccharide (LPS) for 3 days in culture. OA-MSC were isolated of fat tissues obtain from each 3 person with OA. ELISA was performed to measure the levels of IL-6, IL-8, IL-1β, and VEGF (^*^*P* < 0.05, ^**^*P* < 0.01, ^***^*P* < 0.001).

Supplementary Figure 4 Monitoring of OA-MSC time kinetic tracking to joint tissue in MIA induced OA rat. OA rats were injected with VivoTrack 680 NIR Fluorescent Imaging Agent sustained iSTAT3 OA-MSCs. The *in vivo* imaging presented using IVIS Lumina XRMS. After injection of iSTAT3 OA-MSC, the cell remained for 12 day.
