# Supplementary figures and images for: The Therapeutic Effect of STAT3 Signaling-Suppressed MSC on Pain and Articular Cartilage Damage in a Rat Model of Monosodium Iodoacetate-Induced Osteoarthritis
Source: Front Immunol. 2018 Dec 11;9:2881. doi: 10.3389/fimmu.2018.02881 (PMC6305125; doi:10.3389/fimmu.2018.02881)

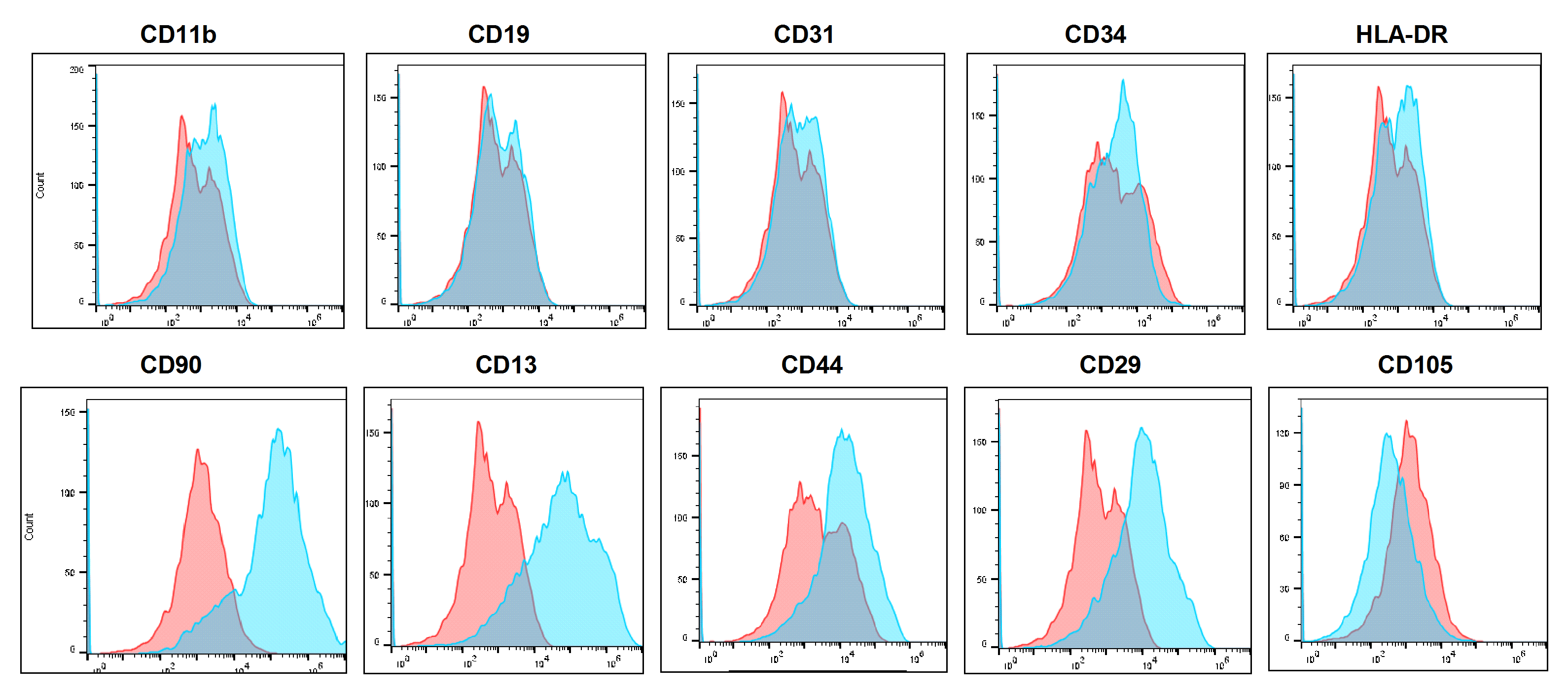

Supplement: Supplementary Figure 1 — Expression levels of MSC surface markers in OA-MSCs. OA-MSCs were isolated from adipose tissue from OA patients. Positive and negative surface markers on OA-MSCs were analyzed by flow cytometry. From left to right: upper row, hematopoietic cell markers CD11b, CD19, CD31, CD34, and HLA-DR; lower row, MSC surface markers CD90, CD13, CD44, CD29, and CD105. Red histograms indicated cells stained with the isotype control; blue histograms indicate cells isolated with specific antibodies. [file Image_1.TIF]

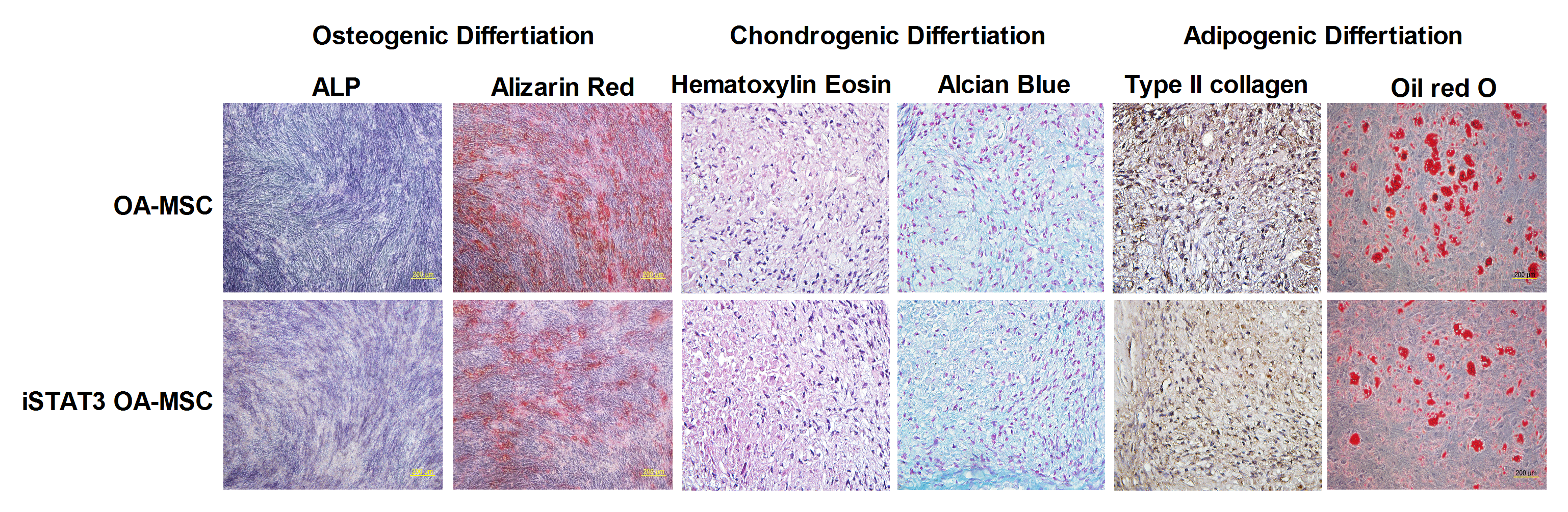

Supplement: Supplementary Figure 2 — Multipotency of OA-MSCs and iSTAT3 OA-MSCs. MSCs can differentiate into a variety of connective tissues cells, including bone, cartilage, and adipose tissue. To observe this multipotent ability, OA-MSCs and iSTAT3 OA-MSCs were plated at 2 × 10^4^ cells/well in a 6-well plate and cultured in osteogenic adipogenic differentiation medium. On day 14, calcium nodule formation was evaluated using Alizarin red and ALP staining, and oil droplets were identified using Oil red O staining. Pellets were cultured to assess MSC chondrogenic differentiation. After 21 days of culture in chondrogenic differentiation medium, pellets were fixed in 10% neutral formalin and embedded in paraffin. The paraffin blocks were sliced at a thickness of 5 μm and stained with H&E, Alcian blue, type II collagen and Oil red O for immunohistochemical analysis. [file Image_2.TIF]

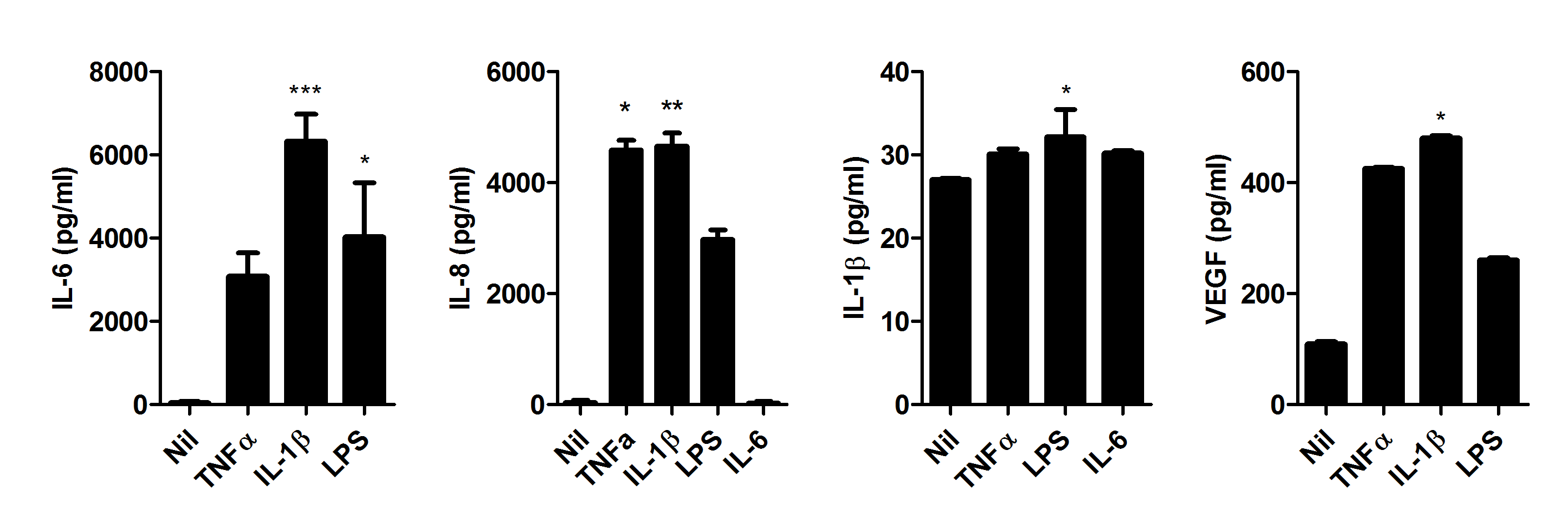

Supplement: Supplementary Figure 3 — Inflammatory cytokine expression by OA-MSCs under inflammatory conditions. OA-MSCs were stimulated by proinflammatory cytokines IL-6, TNF-α, IL-1β, or lipopolysaccharide (LPS) for 3 days in culture. OA-MSC were isolated of fat tissues obtain from each 3 person with OA. ELISA was performed to measure the levels of IL-6, IL-8, IL-1β, and VEGF (*P < 0.05, **P < 0.01, ***P < 0.001). [file Image_3.TIF]

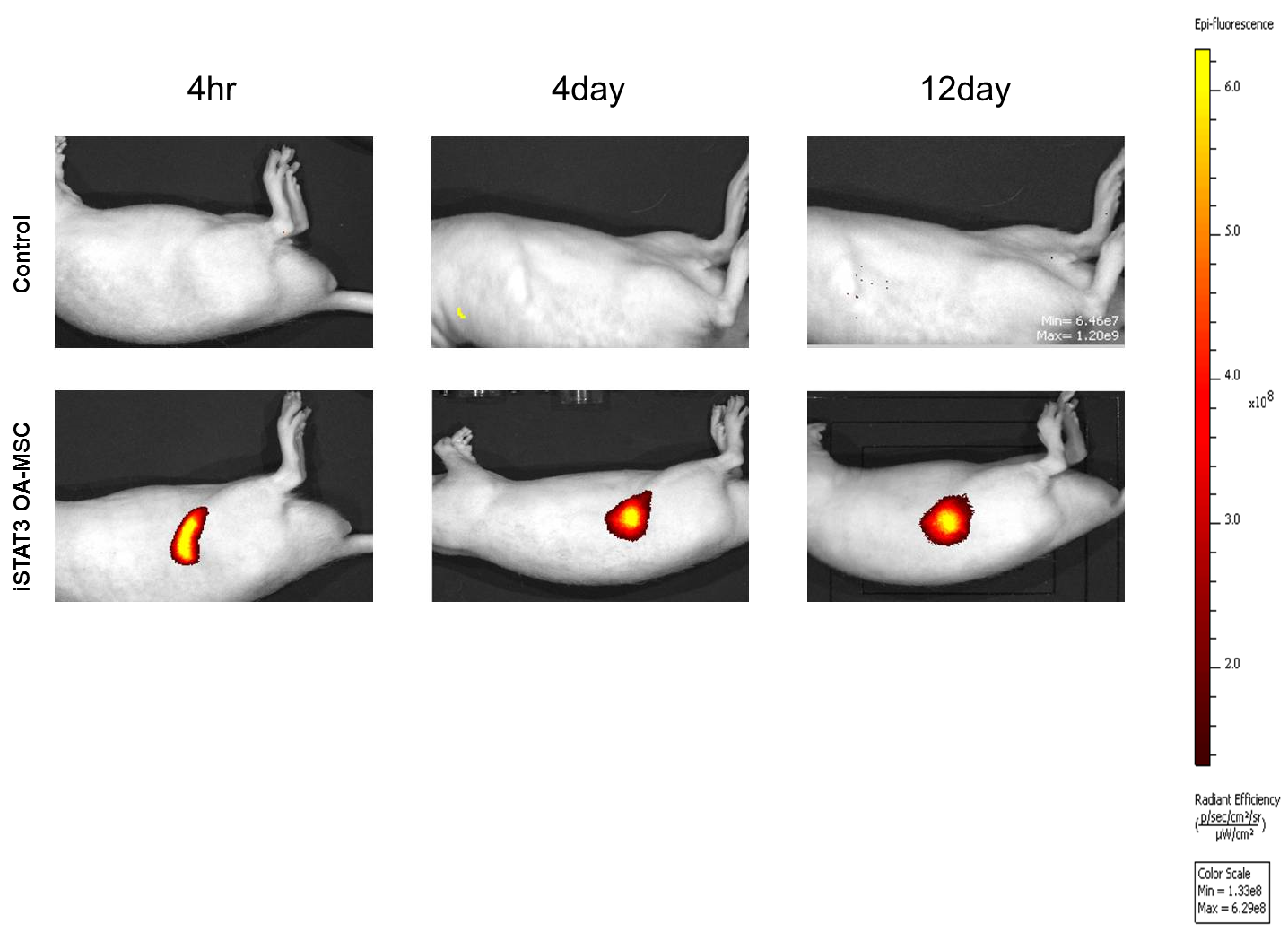

Supplement: Supplementary Figure 4 — Monitoring of OA-MSC time kinetic tracking to joint tissue in MIA induced OA rat. OA rats were injected with VivoTrack 680 NIR Fluorescent Imaging Agent sustained iSTAT3 OA-MSCs. The in vivo imaging presented using IVIS Lumina XRMS. After injection of iSTAT3 OA-MSC, the cell remained for 12 day. [file Image_4.TIF]
